# High Throughput Analysis Reveals Changes in Gut Microbiota and Specific Fecal Metabolomic Signature in Hematopoietic Stem Cell Transplant Patients

**DOI:** 10.3390/microorganisms9091845

**Published:** 2021-08-31

**Authors:** Soumaya Kouidhi, Nessrine Souai, Oumaima Zidi, Amor Mosbah, Amel Lakhal, Tarek Ben Othmane, Dorra Belloumi, Farhat Ben Ayed, Elias Asimakis, Panagiota Stathopoulou, Ameur Cherif, George Tsiamis

**Affiliations:** 1 Laboratory of Biotechnology and Valorisation of Bio-GeoRessources, Higher Institute of Biotechnology of Sidi Thabet, BiotechPole of Sidi Thabet, University of Manouba, Ariana 2020, Tunisia; nessrine.souai@fst.utm.tn (N.S.); oumaima.zidi@hotmail.fr (O.Z.); amor.mosbah@gmail.com (A.M.); ameur.cherif@uma.tn (A.C.); 2 Department of Biology, Faculty of Sciences of Tunis, Farhat Hachad Universitary Campus, University of Tunis El Manar, Rommana, Tunis 1068, Tunisia; 3 Natioanl Bone Marrow Transplant Center, Tunis 1029, Tunisia; lakhalamel@yahoo.fr (A.L.); benothman.tr@gmail.com (T.B.O.); dorrabelloumi@gmail.com (D.B.); 4 Association Tunisienne de Lutte Contre le Cancer (ATCC), Tunis 1938, Tunisia; farhatbenayed@gmail.com; 5 Laboratory of Systems Microbiology and Applied Genomics, Department of Environmental Engineering, University of Patras, 2 Seferi St., 30100 Agrinio, Greece; eliasasim@gmail.com (E.A.); panstath@upatras.gr (P.S.)

**Keywords:** allogenic hematopoietic stem cell transplantation (allo-HSCT), microbiota, metabolomics, biomarkers

## Abstract

There is mounting evidence for the emerging role of gut microbiota (GM) and its metabolites in profoundly impacting allogenic hematopoietic stem cell transplantation (allo-HSCT) and its subsequent complications, mainly infections and graft versus host-disease (GvHD). The present study was performed in order to investigate changes in GM composition and fecal metabolic signature between transplant patients (*n* = 15) and healthy controls (*n* = 18). The intestinal microbiota was characterized by NGS and gas chromatography–mass spectrometry was employed to perform untargeted analysis of fecal metabolites. We found lower relative abundances of Actinobacteria, Firmicutes, and Bacteroidetes and a higher abundance of Proteobacteria phylum after allo-HSCT. Particularly, the GvHD microbiota was characterized by a lower relative abundance of the short-chain fatty acid-producing bacteria, namely, the *Feacalibacterium*, *Akkermansia*, and *Veillonella* genera and the *Lachnospiraceae* family, and an enrichment in multidrug-resistant bacteria belonging to *Escherichia*, *Shigella*, and *Bacteroides*. Moreover, network analysis showed that GvHD was linked to a higher number of positive interactions of *Blautia* and a significant mutual-exclusion rate of *Citrobacter*. The fecal metabolome was dominated by lipids in the transplant group when compared with the healthy individuals (*p* < 0.05). Overall, 76 metabolites were significantly altered within transplant recipients, of which 24 were selected as potential biomarkers. Furthermore, the most notable altered metabolic pathways included the TCA cycle; butanoate, propanoate, and pyruvate metabolisms; steroid biosynthesis; and glycolysis/gluconeogenesis. Specific biomarkers and altered metabolic pathways were correlated to GvHD onset. Our results showed significant shifts in gut microbiota structure and fecal metabolites characterizing allo-HSCT.

## 1. Introduction

Hematopoietic stem cell transplantation (HSCT) is considered to be the most effective form of tumor immunotherapy for many hematologic malignancies and disorders available to date [1]. Although advances in transplant management have greatly improved outcomes, patients, especially those receiving allogeneic hematopoietic stem cell transplantation (allo-HSCT), are at high risk for several complications, such as intestinal inflammation, bloodstream infections (BSI), sepsis, and graft-versus-host disease (GvHD) [2]. Yet early diagnostic tests and biomarkers for prediction remain limited. GvHD, which is a multisystem disorder, remains the major source of morbidity and non-relapse mortality occurring in patients undergoing allo-HSCT [3,4]. However, the etiology of GvHD is not well elucidated. Several studies highlighted the mechanisms of GvHD development, including alloreactive immune cells and pronounced gut microbiota perturbations [5]. In the last decade, the microbiome has gained increasing attention as a crucial factor in allo-HSCT complications. In fact, pre-transplant conditioning includes extensive exposure to chemotherapy, antibiotics treatment, and anti-acid prophylaxis. Added to that, histocompatibility complex differences between the donor and the recipient are factors that may lead to severe damage of the gut epithelia and shifts in the gut microbiota [6,7,8]. Several data have reported that GM diversity and stability decreased by ~30% and could be profoundly related to mortality. During the transplant period, it has been frequently observed that disruption of the gut ecosystem is characterized by the domination of the genera *Enterococcus* or *Streptococcus* [2]. Besides, a decreased abundance of health-promoting species, such as the order Clostridiales, including *Faecalibacterium* and *Ruminococcus*, has been observed [9,10]. As a result, microbiota dysbiosis markedly increases susceptibility to systemic bacterial infections, GvHD onset, and the risk of relapse [11,12]. However, several studies highlighted the importance of metagenomic analysis in finding key bacteria that might solve these complications. In fact, increased amounts of bacteria belonging to another Clostridiales member, the genus *Blautia*, was linked to reduced GvHD lethality in allo-HSCT populations [13]. One of the main mechanisms by which the microbiota influences the host is through its interactions with the host immune system. Hence, a deep exploration of the changes in GM structure is crucial to understanding the crosstalk between GM, immunity, and the intestinal barrier in the genesis of the principal complications following allo-HSCT. Moreover, the modulation of gut microbiota may represent a potential therapeutic intervention to alleviate such complications.

Furthermore, emerging data show that allo-HSCT is followed by major changes in metabolomics profiles that could impact transplant outcomes [14]. However, to date, only a few reports have been published that explore metabolic signature in allo-HSCT. Significant variation of host- and microbiota-derived metabolites that could regulate immunity were identified. Mainly, these metabolites belong to short-chain fatty acids [15]; indole compounds of tryptophan metabolism, a group of ligands for the aryl hydrocarbon receptor (AhR) [16]; and bile acids (BAs) and plasmalogens [14]. Recent experimental studies reported that branched-chain amino acids (BCAA) and butyrate may be potential prediction biomarkers of developing acute GvHD. This suggests that dysbiosis, together with transplantation-related alteration of host metabolism, induces major change in circulating metabolites in recipients that might influence allogeneic immune cell reactivity [14].

However, to date, only a few reports have been published that explore gut microbiota (GM) composition and/or metabolomics in studies involving allo-HSCT. The aim of this study was to characterize for the first time both the fecal microbial structure and metabolomics alterations associated with allo-HSCT and the onset of GvHD.

## 2. Materials and Methods

### 2.1. Study Cohort

With the approval of the ethics committee of Charles Nicolle Hospital and the National Center for Bone Marrow Grafts, we enrolled 15 allo-HSCT recipients for a fecal specimen collection and clinical data study. To be able to investigate the occurrence of GvHD, stool samples were recovered 100 days after allo-HSCT. The subjects provided the fecal specimens within one day of production, and the samples were frozen at −80 °C. Similarly, stool samples were collected from (*n* = 18) healthy subjects and a written informed consent was obtained for each enrollee. All patients underwent either myeloablative (*n* = 12) or nonmyeloablative (*n* = 3) conditioning before transplantation. In addition, patients received grafts from 10/10 HLA-identical siblings. Allo-HSCT recipients were undergoing amoxicillin-based antibiotic treatment prior to allo-HSCT. Patients received antifungal treatment (fluconazol) until day 75 post-graft and an antiviral treatment (valaciclovir) as long as the immunosuppressive treatment (ciclosporin) was maintained. The control group included 9 men and 9 women with an average age of 44 ± 5 years and a BMI of 20 ± 4. Control subjects were healthy Tunisian individuals who were not taking any kind of treatment. Clinical and demographic data of the allo-HSCT recipients and control subjects are listed in Supplementary Table S1. A summary of the high throughput sequencing and alpha diversity analysis outputs are presented in Supplementary Table S2. 

In order to investigate the variability in fecal gut microbiota and whether or not they developed graft-versus-host disease (GvHD), we divided the study cohort into two subgroups, “GvHD” and “no GvHD.” Among the recruited patients in the present study, *n* = 4 patients suffered from acute GvHD, and 11 patients (*n* = 11) were allo-HSCT recipients who did not suffer from GvHD. Age and gender factors were also investigated but not reported in the present study because of non-significance. This could be due to the small number of subjects covering a broad range of ages. 

The study was conducted according to the principles expressed in the Declaration of Helsinki, and all research procedures were approved by the Bioethics Committee of Charles Nicolle Hospital and the National Center for Bone Marrow Grafts.

### 2.2. DNA Extraction, First-Step PCR Amplification, and Purification 

Total genomic DNA was isolated from the fecal specimens using an InnuPREP DNA kit (Analytik Jena, Jena, Germany) according to the manufacturer’s instructions. Three replicates of each sample were extracted. The quality and quantity of DNA samples were tested using a Q5000 micro-volume UV–Vis spectrophotometer (Quawell Technology, San Jose, CA, USA). DNA samples were stored in Eppendorf tubes at −20 °C until PCR amplification and amplicon sequencing analysis. PCR was performed with a KAPA Taq PCR kit (KAPA Biosystems, Wilmington, MA, USA) and the previously extracted DNA as a template. The variable V3–V4 region of the bacterial 16S rRNA gene was amplified with the primer pair U341F-MiSeq and 805R-MiSeq [17]. Each 25 μL reaction contained 2.5 μL of KAPA buffer (10×), 0.2 μL of dNTPs solution (25 mM), 1 μL of each primer solution (10 μM), 0.12 μL of KAPA Taq DNA polymerase solution (5 U/μL), and 1 μL of the template DNA solution, and was finalized with 19.18 μL of sterile deionized water. The PCR amplifications were performed with a 3 min incubation at 95 °C followed by 35 cycles of 95 °C for 30 s, 53 °C for 30 s, and 72 °C for 1 min, and a final 5 min extension at 72 °C. Negative and positive controls were always performed in parallel. All PCR products were separated in a 1.5% (wt/vol) agarose gel in TAE buffer (40 mM Tris-acetate, 1 mM EDTA). The desired amplification product, approximately 550 bp, was visualized in Bio-Rad’s Gel Doc XR+ system. Positive PCR products were purified from unincorporated primers and nucleotides with a 20% PEG, 2.5 M NaCl solution, and centrifuged at 14,000× *g* for 20 min. The precipitate was washed twice with 125 μL of a 70% vol/vol ethanol solution and centrifuged at 14,000× *g* for 10 min. The dried precipitates were suspended in 15 μL of sterile deionized water, and the concentration was measured with a Q5000 micro-volume UV–Vis spectrophotometer (Quawell Technology, San Jose, CA, USA). 

### 2.3. Second-Step PCR Amplification (Indexing) and Purification

The resulting PCR amplicons were diluted up to 10 ng/μL and then used as templates within the second-step PCR for further amplification, and to include the indexes (barcodes) as well as the Illumina adaptors. 

In more detail, the amplification reaction was performed using the KAPA HiFi HotStart PCR kit in a final volume of 50 μL. Each reaction contained 10 μL of KAPA HiFi Fidelity buffer (5×), 1.5 μL of dNTPs solution (10 mM each), 5 μL of the forward indexing primer (10 µ), 5 μL of the reverse indexing primer (10 µM), 1 μL of KAPA HiFi Hot Start DNA polymerase (1 U/μL), 2 μL from the diluted PCR product (10 ng/μL), and 25.5 μL of sterile deionized water. The PCR amplifications were performed with a 3 min incubation at 95 °C followed by eight cycles of 95 °C for 30 s, 55 °C for 30 s, and 72 °C for 30 s, and a final 5 min terminator reaction at 72 °C. The resulting amplicons from the indexing PCR were purified using the NucleoMag NGS Clean-up and Size Selection kit (Macherey-Nagel, Deuren, Germany) according to the manufacturer’s recommendations. Amplicons from different samples were quantified with a Quawell Q5000 micro-volume UV–Vis spectrophotometer and merged in equimolar ratios (8 nM). The combinatorial use of index primers resulted in unique samples that were sequenced by Macrogen using a 2 × 300 bp pair-end kit on a MiSeq platform. 

### 2.4. Bioinformatic and Statistical Analysis

Raw sequencing reads were de-multiplexed and converted to FASTQ, and the Illumina adapters were trimmed using Illumina standard algorithms. Paired-end reads were assembled, trimmed by length, and further corrected for error and quality using the usearch -fastq_mergepairs option. All subsequent analyses were conducted in usearch version v.10 [18]. Briefly, the quality of the assembled sequences was further improved using the -fastq_filter, followed by finding unique read sequences and abundances by using the -fastx_uniques option. Sequences were clustered into operational taxonomic units (OTUs) using the cluster_otus command [19]. Chimeras were removed using the -unoise3 option of usearch v.10 [20]. Taxonomy was assigned using the syntax option against the SILVA 128 release database [20,21]. 

Relative abundance plots were created based on the OTU table along with the taxonomy data previously obtained with the aim of visualizing the most dominant OTUs in each sample group at the phylum and genus levels. Alpha diversity was then computed based on the MicrobioSeq package to analyze within-group diversity [22]. Significant differences between groups were calculated using pairwise ANOVA [23]. Bar charts and boxplots describing alpha diversity and relative abundance were performed using the ggplot2.3.3.2 (RStudio, Houston, TX, USA) package in R version 4.0.3 (The R Foundation for Statistical Computing, Vienna, Austria). Serial group comparison analysis was performed to calculate variables among the study groups and to detect differences in composition and abundance using the non-parametric Kruskal–Wallis rank-sum test along with the Wilcoxon rank-sum test [24].

Principal coordinate analysis (PCoA) was performed on the resulting distance matrix to search for similarities between control and allo-HSCT groups. To estimate the variation of gut bacteria among subgroups depending on the presence or absence of graft-versus-host disease (GvHD) and no-GvHD subgroups compared to healthy controls, a canonical analysis of principal coordinates (CAP) using PERMANOVA+ [25] was performed using a Bray–Curtis similarity index with 999 permutations with a significance of 0.05 applied. All tests were performed using PRIMER 6+ software and the PERMANOVA+ version of this program [25]. Phylogenetic entropy accounting for the relative abundance of OTUs using Allen’s index [26], which is a phylogenetic extension of Shannon’s taxonomic entropy index, was calculated. Allen’s index was computed using an R-function (https://github.com/marlenec/chao, accessed on 20 May 2021, q = 1) that is based on the entropart R-package [27]. Allen’s index increases when the most abundant OTUs are phylogenetically distant.

Furthermore, we aimed to investigate possible interactions between microorganisms. These interactions were analyzed and visualized through the co-occurrence analyses and association network. This may correspond to microorganisms performing similar or complementary functions and/or sharing similar preferred environmental conditions, but not necessarily having physical interactions [28,29]. Co-occurrence network analysis of the main OTUs was performed using the CoNet plugin [30] in Cytoscape 3.8.1 (Institute for Systems Biology, Seattle, WA, USA), and co-occurrence profiles were obtained using Gephi 0.9.2 (Gephi, WebAtlas, Paris, France). To build the network, an ensemble of the Pearson and Spearman correlation coefficients, Mutual Information, and the Bray–Curtis and Kullback–Leibler dissimilarity indices were combined. To compute the statistical significance of the copresence/mutual exclusion, edge-specific permutation and bootstrap score distributions were calculated with 1000 iterations. Edges with original scores outside the 0.95 range of their bootstrap distribution were discarded, and *p*-values were multiple-testing corrected using the Benjamini–Hochberg method. Nodes in each network visualization correspond to microbial OTUs and edges to the microbial associations. The size of each node is proportional to the degree of interactions. 

### 2.5. GC-MS Sample Preparation and Metabolite Extraction

Before GC-MS analysis, QCs were generated by mixing small aliquots of all fecal samples for each group. Blank samples (ethanol and diethyl ether) and QCs were injected into every eighth sample during acquisition.

Fecal water was extracted by performing a successive two solvent-based (ethanol and diethyl ether) metabolite extraction protocol. It was reported in a recent study that extraction efficiency was high for ethanol or methanol protocols [31,32]. The fecal samples were thawed, and 3 g of each stool specimen were mixed with pre-cooled ethanol (−20 °C) in the ratio of 3:20 (g: mL; feces: ethanol). The mixtures were homogenized by sonication for 30 min and centrifuged at 4000 rpm for 20 min at 4 °C. The supernatants were then transferred and filtered through a 0.45 µm Millex-GV syringe filter. A second extraction was conducted by adding 20 mL of diethyl ether to the pellets. The mixtures were well shaken, vortexed for 3 min, centrifuged at 4000 rpm for 20 min at 4 °C, and then filtered through a 0.45 µm Millex-GV syringe filter. All filtered fecal water samples were divided into 150 µL aliquots per Eppendorf tube and dried of solvents, under reduced pressure in a speed vacuum at 10 °C, to form a pellet of concentrated metabolites. All the extracted metabolites were stored at −20 °C until analysis. For the first step, 2 mg ± 0.01 of each sample pellet were grated and diluted with 500 µL of extraction solvent. The mixtures were filtered through a 0.45 µm Millex-GV syringe filter. All samples were run on GC-MS with a 500 µL blank of each extraction solvent. The second step was the derivatization: 2 mg ± 0.01 of each sample were derivatized by adding 800 µL of N-Hexane and 400 µL of (1 M) sodium methylate to the metabolite pellets. The resulting solution was then vortexed, 200 µL of H_2_SO_4_ (0.1 M) was added, and the mixture was homogenized. A total of 500 µL of the supernatants was transferred to GC-MS glass vials. A blank with MilliQ water was prepared and treated the same as the derivatized samples. It is important to note that the same protocol was applied exactly for all the tested samples. A triplicate was performed for each sample. We assessed the relative and absolute abundance of chromatographic peaks in the quality control samples, as described previously [33]. 

### 2.6. GC-MS Analysis and Metabolite Detection

The samples were analyzed using the Agilent GC 7890B MS 240 ion trap gas chromatography technology equipped with an MS detector (GC-MS). Injections were in a splitless mode for 0.75 min, using a 2 mm I.D. non-deactivated direct liner. The separation was carried out on an HP-5MS capillary column (30 m × 0.250 mm; 0.25 μm film thickness). The analysis was carried out in full scan mode for 60 min. The autosampler injected 1 µL of each sample and the separation was performed using the column in split mode and an ionization range from 50 to 1000 mV. The carrier gas was helium with a flow rate of 1.1 mL/min. The injector temperature was set at 280 °C and the GC oven temperature was programmed at 40 °C for 2 min, and then a slope at 50 °C up to 250 °C was maintained for 20 min. The analysis was carried out in full scan mode for 60 min.

### 2.7. Identification and Comparison of Volatile Compounds

Mass spectral data processing and metabolite identification were performed using the Automated Mass Spectral Deconvolution and Identification System (AMDIS) (version 2.71, 2012) and the National Institute of Standards and Technology (NIST) (version 2.0, 2011) database. The detected metabolite peaks were identified using three components within NIST; they were a match of >800, a 90% probability of a match to NIST library standards, and a head-to-tail comparison of the fragments. Metabolite validation was performed by matching experimental tandem MS spectra, retention time, and the CAS number of the metabolic features against the PubChem library (https://pubchem.ncbi.nlm.nih.gov/, accessed on 20 May 2021) as well as the spectral database Human Metabolome Database (HMDB) (https://hmdb.ca/, accessed on 20 May 2021). A compound was present when it satisfied these 3 criteria. This process provides a relative ion abundance; no units of ion abundance are available. A compound with a similarity index of more than 80% was considered as a potential biomarker; therefore, compounds that were found in less than 20% of the entire sample cohort were removed from further analysis [34].

### 2.8. Data Analysis

We attempted to analyze metabolic profiling with the two groups by performing multivariate statistical analysis using SIMCA 16. Initially, principal component analysis (PCA) was carried out to identify any outliers within the data set. Then, an orthogonal partial least squares-discriminant analysis (OPLS-DA) was applied to optimize the separation between the different groups. The model robustness was evaluated with the R²Y (fraction of variance), the Q² (model predictability), and *p*-values. Close to 1, R^2^Y and Q^2^ values indicate an excellent model, whereas low values are indicative of model over-fitting. The variable importance in the projection (VIP) values of all peaks from OPLS-DA were taken as coefficients for peak selection to find the features significantly differentiating between HSCT patients and control groups (CT). The statistical model was tested for robustness by a Y-permutation performed on PLS-DA, which confirmed the observed metabolic variations. The statistical model was tested for robustness by a Y-permutation performed on PLS-DA, which confirmed the observed metabolic variations and by using a CV-ANOVA from SIMCA-P 16 (analysis of variance in the cross-validated residuals of a Y variable). A hierarchical cluster analysis heatmap was obtained using the ward clustering algorithm and Euclidean distance calculation to further confirm the results of PLS-DA and to show the distribution of metabolites among all individuals. The heatmap was performed using the Euclidian distance measurement of similarity followed by a clustering algorithm based on Ward’s linkage, which clusters data to minimize the sum of squares of any two clusters. Analyses were performed using MetaboAnalyst v 5.0.

### 2.9. Selection of Biomarkers

Receiver operating characteristic (ROC) curves were constructed to check the accuracy of the model using MetaboAnalyst v 5.0. A forward stepwise logistic regression model was constructed to design the best metabolite combination. ROC curves were used to test the accuracy of the model. The global performance of each biomarker was evaluated using the area under the curve (AUC) and the determination of sensitivity and specificity [35]. The data obtained were subjected to an unpaired non-parametric test (Wilcoxon rank-sum test, also known as the Mann–Whitney U-test) within MetaboAnalyst and false discovery rates (FDR), calculated by SAM (significant analysis of microarray). The latter analysis is essentially used for microarray data but is also used for metabolomic data (GC-MS, LC-MS, and NMR compounds). These tests were calculated to discover whether selected metabolites were significantly different between groups [36]. All features with FDR values below 0.05 indicated that these features can indeed be regarded as potential “biomarkers”. A metabolomic pathway analysis (MetPA) [37] was applied by MetaboAnalyst v 5.0 to the selected biomarkers to find the most relevant pathways involved in bone marrow transplantation. The area of the circles is proportional to the effect of each pathway, with the color denoting the significance, from the highest in red to the lowest in white.

The difference between GvHD, no GvHD, and healthy controls was calculated with paired-samples *t*-test (Excel 2019). A *p*-value below 0.05 was considered to be statistically significant.

## 3. Results

### 3.1. Hematopoietic Stem Cell Transplant Cohort 

Allo-HSCT individuals were divided into 3 women and 12 men aged between 12 and 62 years. A total dataset of 2,978,313 reads was obtained. The results reported herein are based on the computation of amplicon sequencing data obtained from the 15 patients’ fecal specimens and 18 healthy samples. 

We subdivided our patient cohort into subgroups according to the presence or absence of graft-versus-host disease (GvHD and no-GvHD groups, respectively). Age and gender were also considered but they were not found to be major factors significantly highlighting the shift in the gut bacteria following bone marrow transplantation. Overall, we found five main phyla that were abundant in the study groups, namely, Bacteroidetes, Firmicutes, Proteobacteria, Verrucomicrobia, and Actinobacteria. As for the bacterial genera with the highest abundance in this cohort, we mainly found *Bacteroides*, *Escherichia-Shigella*, and *Faecalibacterium.*

### 3.2. Structure of the Gut Bacterial Community of the Hematopoietic Stem Cell Transplant Recipients 

**Figure 1:**
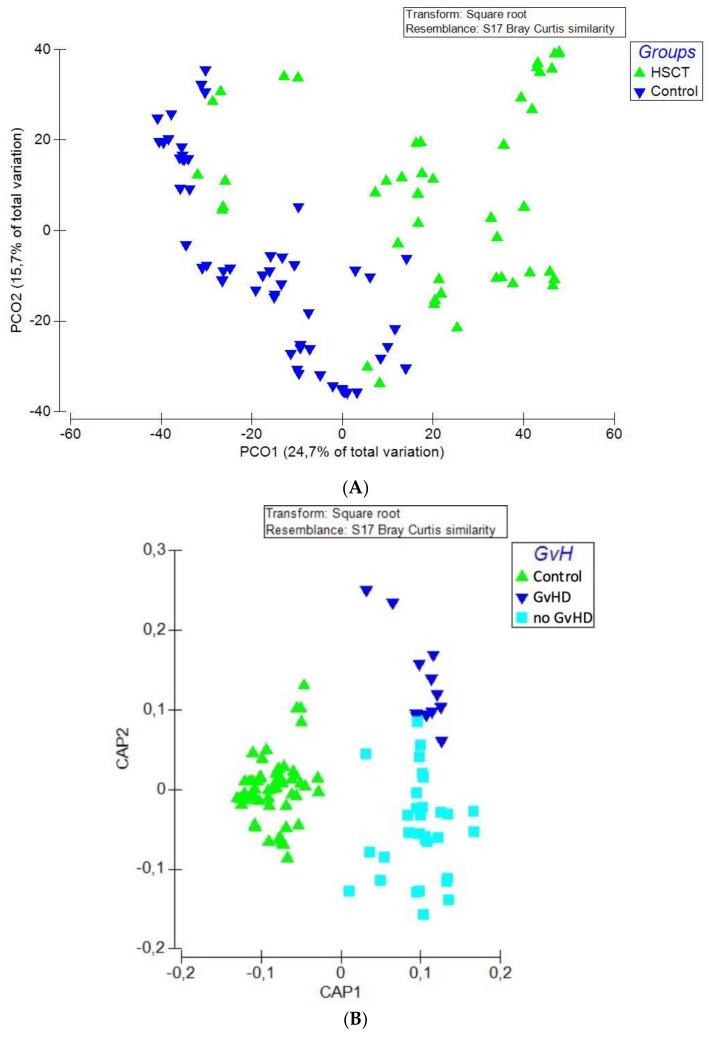
Beta diversity analysis of study groups; (**A**) PCoA plot of the allo-HSCT group (dark blue triangles; *n* = 15) and the control group (green triangles; *n* = 18). (**B**) CAP plot of patient groups depending on the occurrence of graft-versus-host disease (GvHD) (dark blue triangles; *n* = 4), no GvHD (light blue squares; *n* = 11), and control (green triangles). The data matrix was pre-treated by square-root transformation followed by resemblance analysis based on a Bray–Curtis similarity calculation (*p* = 0.001).

**Figure 2:**
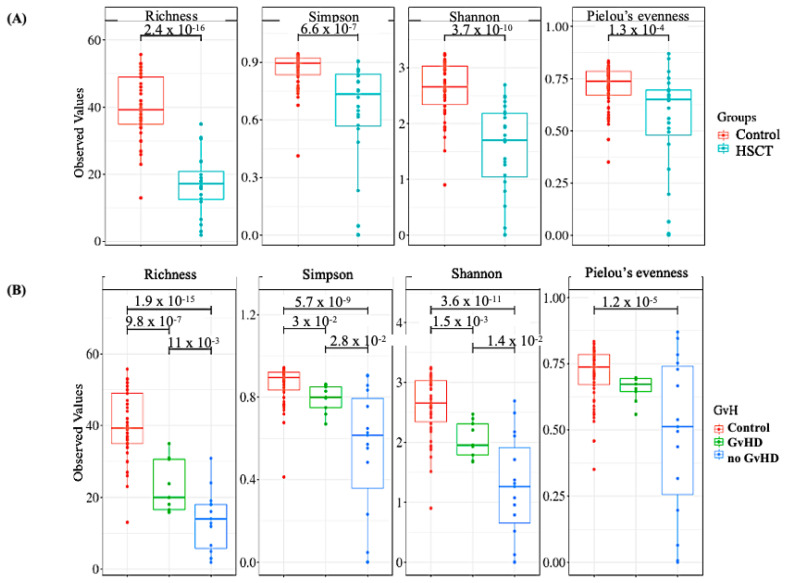
Boxplots of OTU’s richness, Simpson, Shannon, and Pielou’s evenness diversity indexes distinguishing between the hematopoietic stem cell transplant group (allo-HSCT group) and the control group. Statistical analysis was performed with the Wilcoxon rank-sum test. (**A**) Alpha diversity indexes within the control (*n* = 18) and the allo-HSCT group (*n* = 15). (**B**) Alpha diversity analysis among allo-HSCT patients’ richness and diversity indexes within patients with and without graft-versus-host disease (GvHD and no GvHD, respectively) groups (*n* = 4 and *n* = 11, respectively). Study groups are statistically different (*p*-value < 0.05; Wilcoxon rank-sum test).

The bacterial community in hematopoietic stem cell transplant (allo-HSCT) patients was significantly different compared to healthy subjects. Beta diversity analysis (inter-group diversity) was performed using principal coordinate analysis (PCoA) using the unweighted Unifrac metric of fecal microbiota among all samples. The allo-HSCT group was found to be significantly different than the control group (*p* = 0.001, resemblance test/Bray–Curtis similarity), as shown by PCoA analysis (Figure 1A). Alpha diversity (within-sample diversity) analysis showed significant difference in both microbial diversity and richness between study groups. In fact, the median Shannon diversity index was lower in the allo-HSCT fecal specimens than in the control group (1.7 vs. 2.7, respectively, *p* = 3.7 × 10^−10^, Wilcoxon rank-sum test) (Figure 2A). Meanwhile, a higher median richness index was noticeable in the control group than in the allo-HSCT group (50 vs. 18, respectively, *p* = 2.4 × 10^−16^, Wilcoxon rank-sum test) (Figure 2A). The overall variation between study groups could be explained by a bacterial dysbiosis that was induced by the disease state on one hand and by the allo-HSCT outcomes on the other hand when compared to the healthy state.

At the phylum level, Bacteroidetes (mean ± SE; 57.61% ± 2.86% vs. 39.60% ± 3.88%) and Firmicutes (34.41% ± 2.44% vs. 17.68% ± 2.67%) were the most dominant members of the gut microbiota present in the fecal specimens in control and allo-HSCT patients. Additionally, bacterial profiles showed the presence of the Actinobacteria phylum with higher relative abundances among the control individuals than in the patient group (mean ± SE; 1.97% ± 0.62% vs. 0.28% ± 0.11%). Interestingly, Proteobacteria and Verrucomicrobia were found to be more abundant in the allo-HSCT group (33.95% ± 4.66% vs. 4.29% ± 1.07% and 4.90% ± 2.53% vs. 1.43% ± 0.57%, respectively) (Supplementary Figure S1A). 

**Figure 3:**
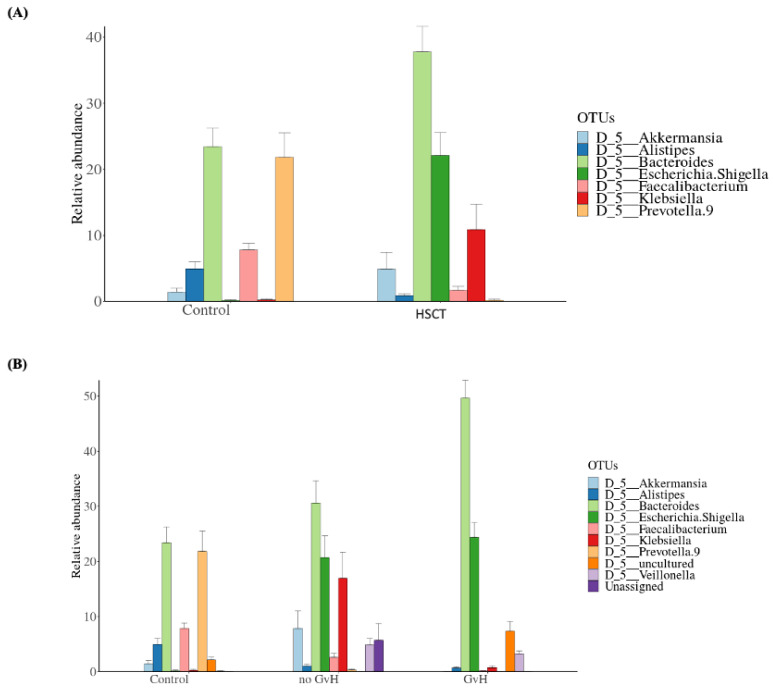
Comparison of the mean relative abundance of bacterial operational taxonomic units (OTUs) at the genus between (**A**) the control group (*n* = 18) and allo-HSCT recipients (*n* = 15), and (**B**) control subjects; patients suffering and non-suffering from graft-versus-host disease (*n* = 4 and *n* = 11, respectively).

Evaluation of the most common taxa (>1% mean relative abundance among all fecal specimens) was performed at the genus level. The relative abundance of *Faecalibacterium*, *Alistipes*, and *Prevotella* 9 was significantly lower and the relative abundance of *Bacteroides*, *Escherichia*/*Shigella*, *Klebsiella*, and *Akkermansia* was higher in the allo-HSCT fecal specimens than in the fecal specimens from the control group (Supplementary Table S3). A bar chart of the seven most abundant genera is shown, with three genera being lower in the allo-HSCT fecal specimens and four genera being higher in the patient group compared to the fecal specimens from the control group (Figure 3A). 

Serial group comparison analysis enabled us to identify four genera that were significantly different between the allo-HSCT and control groups. These genera were *Escherichia*/*Shigella*, *Faecalibacterium*, *Bacteroides*, and *Prevotella* 9 (Adj-*p*-values: *p* = 0; 0; 0.046; and *p* = 10^−4^, respectively, Wilcoxon rank-sum test) (Supplementary Figure S2). 

### 3.3. Bacteriome of Graft-Versus-Host Disease GvHD

Beta-diversity and alpha diversity analysis were performed to investigate differences in whole gut microbial diversity in patients with and without graft-versus-host disease (GvHD) after samples from allo-HSCT patients were compared to healthy controls (Figure 1B and Figure 2B).

Relative abundance analysis showed that the phylum Bacteroidetes had a lower relative abundance in the patient groups with GvHD (GvHD subgroup) compared to patients not suffering from GvHD and to healthy controls (mean relative abundance ± SE; “Control” 1.43% ± 0.57% vs. “GvHD” 0% ± 0% vs. “no GvHD” 7.84% ± 3.17%). 

The evaluation of the most common taxa at the genus level showed a higher relative abundance of *Klebsiella*, *Akkermansia*, and *Veillonella* in the samples from the no-GvHD subgroup, including individuals not suffering from graft-versus-host disease, whereas the genera of *Escherichia/Shigella* and *Bacteroides* were highly abundant in the subgroup of patients with graft-versus-host disease (GvHD) (Figure 3B). As for *Prevotella 9*, *Alistipes*, and *Faecalibacterium*, these genera were more abundant among control individuals (Supplementary Table S4 and Figure 3B).

Serial group comparison analysis was performed in order to detect significant differences in taxa composition and abundances between the mainly GvHD and no-GvHD groups. Similar to the relative abundance results, we identified the *Escherichia/Shigella* and *Bacteroides* genera as having significant differences between the two patient subgroups (Adj-*p*-values = 0 and 0.01, respectively). Furthermore, this analysis enabled us to identify an additional OTU that was significantly more abundant in the no-GvHD group, namely, *Bacterium NALE-ZL*, belonging to the *Lachnospiraceae* family of the Firmicutes phylum (Adj-*p*-value = 0.0056). Serial group comparison was calculated using the Wilcoxon rank-sum test (Supplementary Figure S3).

Phylogenetic entropy varied significantly among the control and the GvHD and the no-GvHD groups (Supplementary Figure S5) (Kruskal-Wallis test performed on Allen’s index of the three groups examined, *p* = 0.007). 

**Figure 4:**
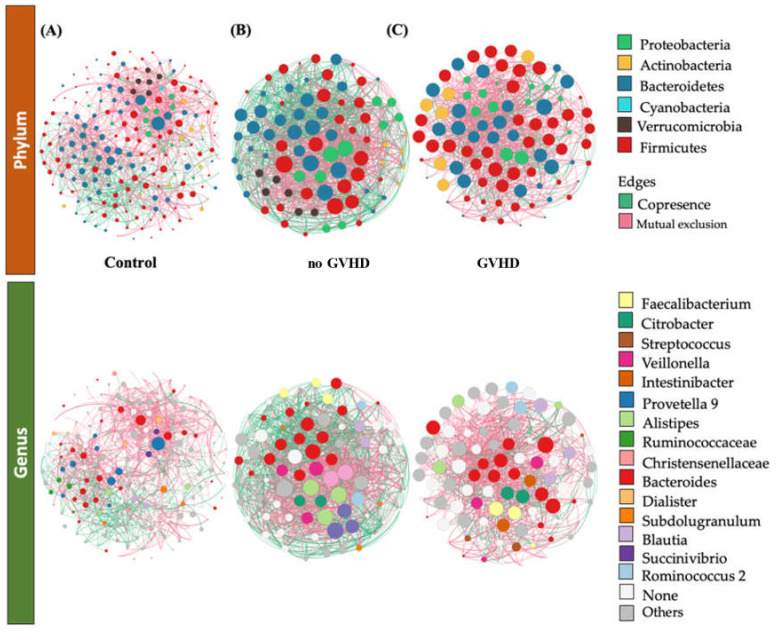
Significant co-occurrence (red edges) and mutual exclusion (green edges) network analysis. Interactions among OTUs in (**A**) control group (*n* = 18), (**B**) GvHD patient group (*n* = 4) and in (**C**) the no GVHD subgroup (*n* = 11). For each group, the nodes correspond to the present OTUs colored according to phylum or genus affiliation. The size of the nodes is proportional to their degree of connection (the number of edges associated with the node).

To identify potential interactions and niche-sharing among bacterial partners, we constructed inter-kingdom co-occurrence and mutual exclusion networks related to the allo-HSCT patient subgroups (with and without GvHD) and healthy subjects. The topological properties are shown in Supplementary Table S5. The two networks were visualized at both the genus and phylum levels and found to exhibit significantly different structures (Figure 4A–C). Although, the number of nodes in allo-HSCT recipients with GvHD and no GvHD was not variable (90 and 83, respectively), both subgroups showed a significantly lower interaction rate compared to control subjects (194). Meanwhile, the number of edges was found to be higher within the GvHD subgroup than control subjects and the no-GvHD subgroup, namely, 1885 vs. 1415 and 1416, respectively. Network analysis also indicated a higher clustering coefficient within the GvHD subgroup (0.667 vs. 0.270 and 0.487, respectively). This indicates that the network structures of gut communities are significantly altered in the GvHD subgroup compared to healthy subjects and the subgroup of patients with no GvHD (Supplementary Table S5, Figure 4). Since the size of the nodes is proportional to their degree of connection, network visualization suggests that Bacteroidetes and Firmicutes possess a higher degree of interaction among all bacterial phyla in the gut among all groups, along with Proteobacteria and Actinobacteria in the allo-HSCT subgroups (Figure 4 and Supplementary Figure S4). A closer look at the profile of hub bacterial genera (genera showing the highest number of significant positive and/or negative correlations with other members of the community) revealed that in the gut microbiota of control individuals, the genera *Bacteroides* and *Prevotella* 9 had the highest number of connections (mostly mutual exclusion) with the rest of the community. As for the no-GvHD subgroup, the genera *Klebsiella* and *Veillonella* had the highest number of mutual exclusion relationships, whereas the genera *Bacteroides*, *Feacalibacterium*, and *Alistipes* had higher copresence interactions. Meanwhile, network analysis of gut microbiota among the GvHD subgroup showed that the genera *Bacteroides* and *Citrobacter* had the highest number of connections (mutual exclusion relationships), along with *Blautia*, which had the highest copresence interaction rate within the rest of the bacterial community in the GvHD subgroup (Supplementary Figure S4).

### 3.4. Metabolomics Profiling of Patients after allo-HSCT

Herein we studied two cohorts, one (allo-HSCT cohort, *n* = 15) from allo-HSCT patients and one from 18 healthy controls. Fecal samples from patients were obtained after stem cell transplantation. Importantly, almost all the subjects in our study had a balanced diet prior to sampling to exclude the possibility that metabolic changes might be attributed to nutrition differences. Fecal samples were analyzed using a GC-MS approach that has been shown to produce a comprehensive metabolic fingerprint with good analytical characteristics in fecal samples and is considered a suitable tool to investigate the metabolic abnormality following hematopoietic stem cell transplantation. In the above-described GC-MS analyses, we detected about 175 metabolites. Significant differences in the total ion chromatogram of the fecal samples were clear between allo-HSCT recipients and healthy subjects, as shown in Supplementary Figure S6. The metabolites were identified after being handled with the NIST’s three criteria: a match of >800, a 90% probability of a match to NIST library standards, and a head-to-tail comparison of the fragments. After excluding the non-endogenous metabolites (such as drugs, solvents, and reagents) and those with missing values, a total of 76 metabolite features (see Supplementary Table S6) were detected, including both hydrophilic and hydrophobic metabolites.

The average distribution of these shared metabolites into each metabolic pathway was lipids (42%), hydrocarbons (13%), benzoids (7%), dicarboxylic acids (7%), terpenoids (5%), carboxylic acids (4%), indoles (3%), phenols (3%), aldehydes (3%), vitamins (3%), diamines (1%), furans (1%), ketones (1%), food additives (1%), naphtalenes (1%), and other metabolites (5%) (Supplementary Figure S7). All 76 metabolite features were used for the following multivariate (OPLS-DA) statistical analysis.

### 3.5. Alteration of Patients’ Metabolome Compared to Healthy Subjects

**Figure 5:**
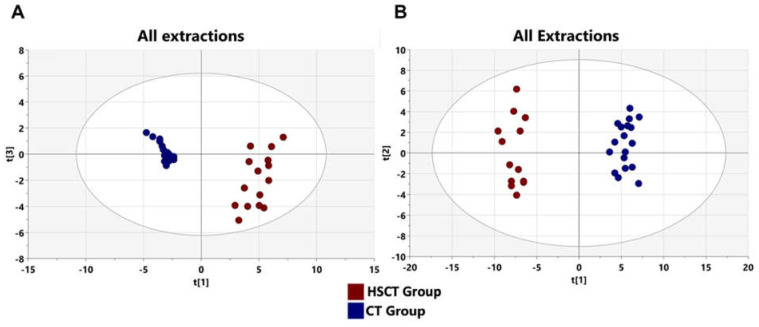
PCA and OPLS-DA score plots of the fecal metabolic profiles from the (allo-HSCT) and control (CT) groups. An overview of the data from the two extractions confirms that there are no outlying samples within a 95% confidence interval. (**A**) PCA score plot model with R^2^(X) = 0.632 and Q^2^ = 0.557 values. Red circles represent allo-HSCT samples and blue circles represent CT samples. (**B**) Orthogonal partial least squares-discriminant analysis (OPLS-DA) score plot model showing separation based on all extraction methods with R^2^(X) = 0.482, R^2^(Y) = 0.981, Q^2^ = 0.909 and cross validated analysis of variance (CV-ANOVA) *p* = 3.956 × 10^−12^ values. Blue circles represent healthy control samples and red circles represent allo-HSCT samples.

To determine whether the metabolic profile of hematopoietic stem cell transplant patients differs from that of healthy subjects, an unsupervised PCA was initially used to generate an overview of variations between groups and to identify the existence of outliers. As we can see in Figure 5A, the PCA model showed a clear trend of group clustering between the bone marrow (allo-HSCT) transplant group and the control healthy (CT) group, with an absence of outliers. To better show the significant fecal metabolic differences between the allo-HSCT and CT groups, a supervised OPLS-DA was conducted with three orthogonal and one predictive component calculated for the model. The metabolomic signature showed dramatic changes. The score plots between the CT and allo-HSCT groups in the feces showed clear profile separation between both groups. The two-dimensional (2D) (Figure 5B) OPLS-DA score plots of fecal metabolite profiling among the CT and allo-HSCT groups showed that the two groups could be distinguished clearly by fecal metabolite profiling with good model fitness and predictability (R^2^X = 0.482; R^2^Y = 0.981, and Q^2^ = 0.909).

To validate the model, a permutation test with *n* = 200 was performed (Supplementary Figure S8). Furthermore, a CV-ANOVA test was performed to check the statistical significance of the differences between the two groups in the OPLS-DA model, which resulted in a score of *p*-value = 3.956 × 10^−12^, indicating that the differences between the groups within the model were highly significant.

**Figure 6:**
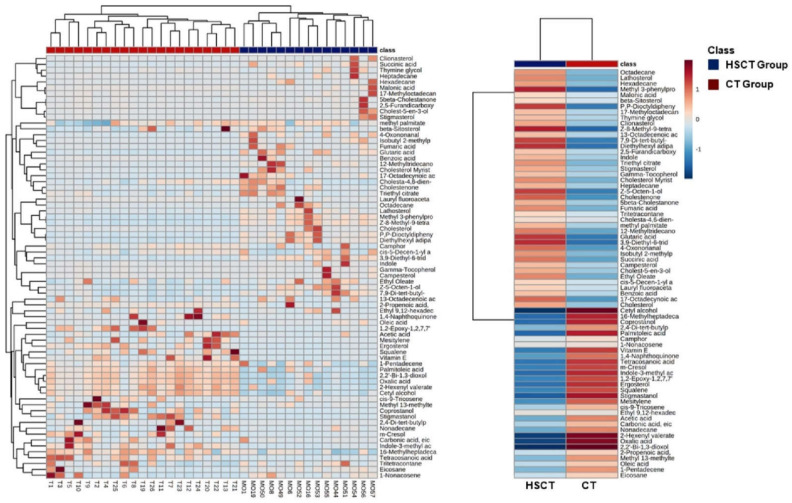
Fecal metabolic differences among groups: heatmaps of differential metabolites among the groups. The color of each section represents the significance of the change in metabolites (red: upregulated; blue: downregulated). Rows: metabolites; columns: samples.

Fluctuations of the different metabolites between the two groups were investigated and the results are exhibited in the heatmaps shown in Figure 6. The relative intensity of most of the hydrocarbons (eicosane; nonadecane, 1-pentadecene; carbonic acid, eicosyl vinyl ester; 1-nonacosene and Cis-9-Tricosene), vitamin E, phenols, food additives, naphthalenes, m-cresol, indole-3-methyl acetate, and terpenoids decreased concentrations in the allo-HSCT recipients compared to healthy subjects. However, most lipids (fatty acids, sterols, fatty alcohols, and steroid derivatives), benzoids, gamma-tocopherol, ketones, indole, furans, diamine, acetic acid, and dicarboxylic acids showed high levels in allo-HSCT recipients compared to the CT group.

### 3.6. Identification of Potential Biomarkers of Allogenic Hematopoietic Stem Cell Transplant Status and Biological Explanation

To identify potential biomarkers of allo-HSCT patients and investigate transplantation impact on the metabolome profile, relevant metabolites were selected between the control and HSCT groups using VIP values (>1.0) from OPLS-DA and FDR (<0.05). Following both ethanol and diethyl ether extractions, a total of 24 differential metabolites (see Table S7) in feces were selected as potential biomarkers of HSCT patients.

**Figure 7:**
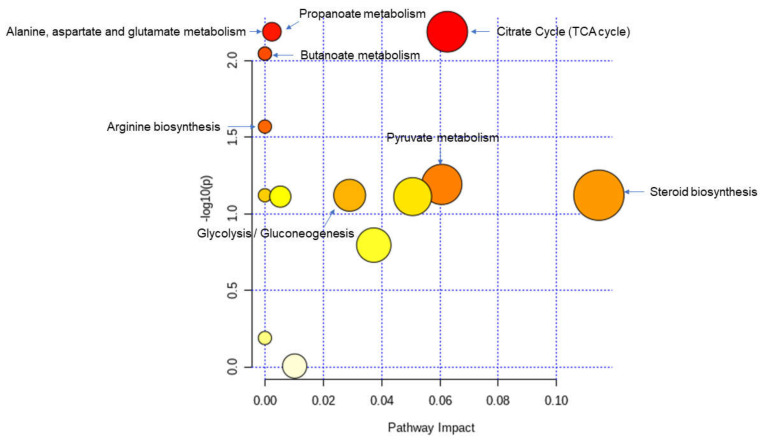
Summary of the pathway analysis with MetPA where all the metabolites were considered. The area of the bubbles is proportional to the effect of transplantation on each pathway, with the color denoting the significance, from highest in red to lowest in white.

All the altered metabolites were then used to further analyze the differential metabolic pathways in the allo-HSCT patients by MetaboAnalyst v 5.0. The results revealed that the metabolic pathways of the TCA cycle (*p* = 6.47 × 10^−4^); alanine, aspartate, and glutamate metabolism (*p* = 5.21 × 10^−3^); propanoate metabolism (*p* = 5.21 × 10^−3^); butanoate metabolism (*p* = 9.00 × 10^−3^); arginine biosynthesis (*p* = 1.69 × 10^−2^); pyruvate metabolism (*p* = 2.43 × 10^−2^); steroid biosynthesis (*p* = 3.56 × 10^−2^); and glycolysis/gluconeogenesis (*p* = 4.12 × 10^−2^) were altered due to the impact of the transplantation process (Figure 7).

### 3.7. GvHD Onset Is Characterized by Specific Metabolomic Changes

**Figure 8:**
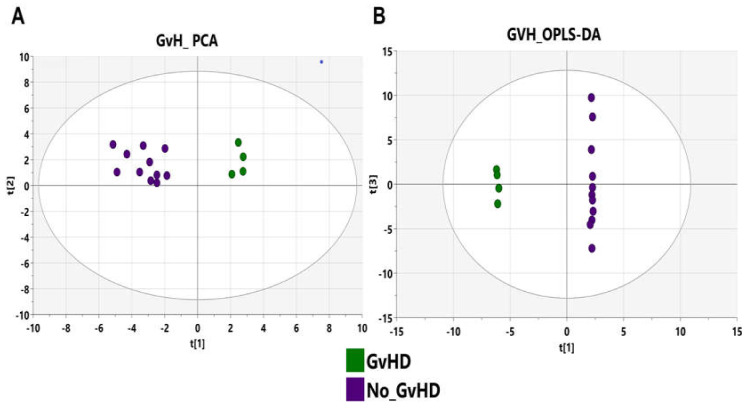
PCA and OPLS-DA score plots of the fecal metabolic profiles from the GvHD and no-GvHD groups. (**A**) PCA score plot model with values of R^2^ = 0.596 and Q^2^ = 0.476. Green circles represent patients who developed GvHD after the graft and purple circles represent patients without GvHD. (**B**) Orthogonal partial least squares-discriminant analysis (OPLS-DA) score plot model showing separation based on the two extraction methods with R^2^(X) = 0.601, R^2^(Y) = 0.974, Q^2^ = 0.923 and cross-validated analysis of variance (CV-ANOVA) *p* = 0.0256 values. Green circles represent GvHD samples and purple circles represent no-GvHD samples.

As the metabolome reflects the metabolism of both host cells and microbiota in humans, we compared the metabolome of patients with GvHD to that of recipients without GvHD to determine metabolomic changes that are associated with GvHD development. First, we investigated the data obtained for age, gender, and body mass index (BMI), as they are considered the main confounding factors, and to confirm that the metabolome changes detected were due to GvHD. An OPLS-DA analysis was performed to investigate each factor previously mentioned. Other than GvHD occurrence, no statistically significant changes related to the previously listed factors were observed (no clear separation in the OPLS-DA with a *p*-value > 0.05). To confirm the implication of GvHD, PCA was used, and the obtained result showed that the GvHD and no-GvHD groups were clearly discriminated, with no outliers (Figure 8A). To better explore the significant fecal metabolic differences, a supervised OPLS-DA was conducted with two orthogonal and one predictive component calculated for the model. The metabolomic signature showed dramatic changes. The score plots showed a clear profile separation between both groups with good model fitness and predictability (R^2^X = 0.601; R^2^Y = 0.974, and Q^2^ = 0.923) (Figure 8B).

To validate the model, a permutation test with *n* = 100 was performed (Supplementary Figure S9). Furthermore, a CV-ANOVA test was performed to check the statistical significance of the differences between the two groups in the OPLS-DA model, which resulted in a score of *p* = 0.0256, indicating that the differences between the groups within the model were highly significant. The metabolites that mostly contributed to the GvHD profile were then identified with ROC analysis (see Table S8) and used to build an overview of the altered pathways. 

**Figure 9:**
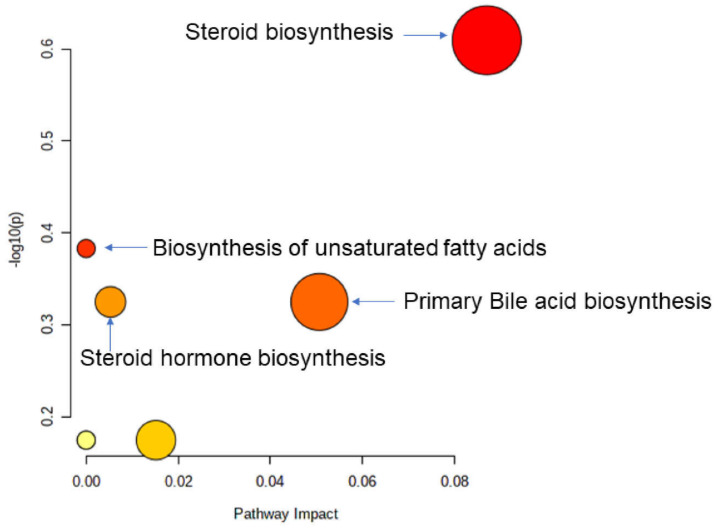
Summary of the pathway analysis with MetPA where all the metabolites were considered. The area of the bubbles is proportional to the effect of each pathway, with the color denoting the significance, from highest in red to lowest in white.

This approach confirmed that the previously identified metabolites, cholesterol, (9Z)-octadecenoic acid, campesterol, and lathosterol, belong to steroid biosynthesis (*p* = 6.07 × 10^−9^), biosynthesis of unsaturated fatty acids (*p* = 2.49 × 10^−4^), primary bile acid biosynthesis (*p* = 3.56 × 10^−3^), and steroid hormone biosynthesis (*p* = 7.47 × 10^−3^) (Figure 9).

In our study, we had four patients with GvHD and 11 patients without GvHD. To investigate the changes in metabolite profile between the two cohorts (GvHD and no GvHD) and the healthy subjects, a t-test was performed for each compound (see Table S9).

Compared to healthy subjects, the allo-HSCT patients with GvHD were mainly characterized by a significant increase in the amounts of some lipid metabolites, such as 17-octadecynoic acid (*p* = 0.0014), 12-methyltridecanoic acid (*p* = 0.0294), methyl 3-phenylpropanoate (*p* = 0.026), coprostanol (*p* = 0.0002), lathosterol (*p* = 0.0211), the phenol methyl 3-phenylpropanoate (*p* = 0.0344), and succinic acid (*p* = 0.013), and a significant decrease in 2,4-di-tert-butylphenol (*p* = 0.0231) and acetic acid (*p* = 0.0122). However, the allo-HSCT patients without GvHD were mainly distinguished from the CT group by a significant decrease in the levels of the same lipid metabolites as the GvHD group: vitamin E, indole-3-methyl acetate, 7,9-di-tert-butyl-1-oxaspiro[4 ,5]deca-6,9-diene-2,8-dione, and m-cresol with a *p*-value < 0.05, whereas squalene (*p* = 0.0003), succinic acid (*p* = 0.00038), ergosterol (*p* = 0.0094), and stigmastanol (*p* = 0.00016) exhibited increased amounts compared to the healthy control profile (Supplementary Figure S10).

## 4. Discussion

Allogeneic hematopoietic stem cell transplantation (allo-HSCT) is a major treatment for hematologic malignancies and for inherited or acquired hematopoiesis disorders. However, it is still hampered by high morbidity and mortality rates, mainly due to graft-versus-host disease (GvHD). Several recent studies have demonstrated that patients undergoing allo-HSCT are subjected to extreme shifts and decreased diversity in the intestinal microbiota, which affects immune defenses and host–microbe interactions. Furthermore, emerging data have suggested that metabolomic pathways are influenced by the transplantation process and changes that are more specifically associated with acute GvHD at disease onset.

According to our findings, allo-HSCT allograft is associated with a profound modification of the gut bacterial ecosystem. Compared to healthy microbiota, the intestinal microbiome following HSCT was characterized by a significant decrease in bacterial diversity. The loss of microbiota diversity is linked to an increased risk of infections, GvHD, and mortality after allo-HSCT [7,13,38].

A recent paper by Zama et al. reported that significant shifts in microbiota composition could be identified shortly after transplantation, and that the pre-transplant microbiota differences can be connected to post-transplant events (e.g., infections, GvHD, relapse) [2]. Herein, we studied the differences in gut microbial profiles between an allo-HSCT group, GvHD/no-GvHD subgroups, and healthy controls. The comparison between the healthy microbiome and that of allo-HSCT patients at the phylum level revealed that the allo-HSCT microbiota was found to be dominated by Proteobacteria phylum members, which constituted over 30% of the total microbial population. In line with our results, a 16S rRNA gene analysis by Taur et al. seemed to correlate proteobacterial domination with the increase risk of Gram-negative rod bacteremia five-fold [39]. Moreover, Harris et al. evaluated the microbial community in fecal samples of allogenic hematopoietic SCT (allo-HSCT) and revealed that post-engraftment pulmonary complications and γ-proteobacteria domination were predictive of mortality [40]. We also found a significant decrease in the relative abundance of Bacteroidetes following allo-HSCT. Previous characterization of the fecal microbiota of patients undergoing allogeneic hematopoietic stem cell transplantation by Ubeda et al. demonstrated that intestinal colonization with *Barnesiella*, a member of the Bacteroidetes phylum, confers resistance to intestinal domination and bloodstream infection with vancomycin-resistant *Enterococcus* (VRE) [41]. This result reveals that lower Bacteroidetes can possibly cause post allo-HSCT infections.

Our results show that at the genus level, the genera *Bacteroides*, *Escherichia*/*Shigella*, *Klebsiella*, and *Akkermansia* were higher in the allo-HSCT fecal specimens than in the control group. Consistent with that, a study investigating the gut bacterial composition of allo-HSCT and healthy control groups revealed that allo-HSCT samples had a higher abundance of *Escherichia coli* and *Klebsiella pneumoniae* [42]. They found that patients with *Escherichia coli* and *Klebsiella pneumoniae* bloodstream infections had concomitant gut colonization with these microorganisms, suggesting that the gut may be a source of these infections. These results provide convincing evidence that the gut microbiota is clinically altered after allo-HSCT.

Besides bloodstream infections, GvHD is the main complication of allogenic hematopoietic SCT (allo-HSCT) during the treatment of hematological disorders [43]. A relationship between the microbiota and GvHD has long been suspected but experimental observations are still few, mainly involving cohorts of adult patients [44]. There is growing evidence that the microbiota could impact GVHD, and that GVHD could also lead to dysbiosis of the microbiota [45,46]. Recent studies showed that GVHD deteriorates intestinal barrier function, suggesting that the conditioning regimen that allo-HSCT patients undergo and GVHD can cause synergistic damage to the epithelium [47,48]. In the present study, we successfully demonstrated a different gut microbial structure after allo-HSCT between GvHD and non-GvHD subjects. Our findings suggest that post-allo-HSCT dysbiosis is characterized, at the phylum level, by a lower abundance of Firmicutes. PCR studies after allogeneic stem cell transplantation (ASCT) in 131 patients demonstrated a loss of bacteria in the Firmicutes phylum, and an association of GvHD risk with both relative abundance and prevalence of enterococci [49]. At the genus level, we found that *Faecalibacterium* had lower relative abundance in GvHD patients compared to the no-GvHD and control groups. In mouse models, the reduction of obligate anaerobic bacteria from the Clostridiales order (*Faecalibacterium, Roseburia*, *Ruminococcus*, and *Blautia*) characterized specific dysbiosis associated with GvHD, among others [50,51]. Data found in both human adults and children confirm those found in mouse models. In particular, GvHD onset was associated with a drop in the abundance of the known health-promoting *Faecalibacterium*, namely, butyrate-producing *Faecalibacterium prausnitzii* and high percentages of *Enterococcus* [52,53]. Of note, our study demonstrated an increase in the relative abundance of both *Bacteroides* and *Escherichia/Shigella* genera after GvHD. Some clinical trials have been performed to characterize the gut microbiota after allo-HSCT and found specific GvHD OTUs, including several OTUs representing multidrug-resistant *Bacteroides spp*. and *Escherichia/Shigella sp*. [54].

In this pilot study, a significant decrease in important short-chain fatty acid (SCFA) producers, mainly *Veillonella* and *Feacalibacterium*, was observed in the GvHD group compared to no-GvHD patients and to control subjects. These SCFAs consist of propionate produced by species belonging to the genus *Veillonella* and the histone deacetylase inhibitor butyrate produced by the *Feacalibacterium* genus [55]. Butyrate is a major metabolite in colonic lumen arising from bacterial fermentation of dietary fiber and has been shown to be a critical mediator of the colonic inflammatory response. Butyrate is a source of energy and plays a critical role in homeostasis through its ability to counteract inflammation-mediated ulcerative colitis [56]. Patients receiving allo-HSCT as treatment for hematologic malignancies and who have an abundance of butyrate-producing bacteria have the highest probability of getting rid of carbon dioxide (GvHD), whereas those with a low abundance of butyric acid bacteria belonging to Firmicutes, Ruminococcaceae, and Lachnospiraceae are more often faced with GVHD [12]. In the present study, our serial group analysis enabled us to identify a significantly higher abundance in the no-GvHD subgroup of an OTU belonging to the Lachnospiraceae family compared to the GvHD patients. Consistent with our results, Han et al. reported that the bacterial community after allo-HSCT was depleted of Clostridia (e.g., the Lachnospiraceae and Ruminococcaceae families) and enriched with Gammaproteobacteria (e.g., the Enterobacteriaceae family) in the acute GvHD group compared to the non-acute GvHD group [55]. Moreover, Lachnospiraceae was positively correlated with the Treg/Th17 ratio, suggesting that intestinal microbiota might induce acute GvHD by influencing the Treg/Th17 balance [57]. Our findings demonstrate that the depletion of these important SCFA producers in the gut intestinal tract enhances the risk of developing GvHD after allo-HSCT. Interestingly, we found a predictive GvHD biomarker in the no-GvHD group, namely, the *Akkermansia* genus. These bacteria are known for their mucus-degrading capabilities; recent studies in mice revealed that mucus degradation can contribute to GvHD [58]. Finally, the Actinobacteria phylum was reported to be an important biomarker of survival following allo-HSCT [59]. This phylum was found to be dramatically lower in our cohort of patients compared to healthy subjects.

Other potential biomarkers related to post allo-HSC transplantation dysbiosis are shown through the network analysis and bacterial co-occurrence visualization among members of the gut microbiota. Our interaction network analysis revealed that within the microbiota of stool contents, the occurrence of GvH disease resulted in an increased number of positive interactions of *Blautia* genus with other members of the community despite its low relative abundance. Moreover, the present findings indicated a higher inhibition rate of the Bacteroidetes and Firmicutes phyla over the rest of the community members. Mainly, *Bacteroides* and *Citrobacter* genera had a higher number of mutual exclusion interactions in allo-HSCT transplant recipients suffering from GvH disease than those who were more stable.

*Blautia* has previously been associated with GvHD in only 2 other studies [57,60]; however, there have been associations with GvHD severity [61] and GvHD-associated mortality [13]. Here, we were able to confirm and clarify the association of *Blautia* with the development of GvHD. Moreover, Louis and his coworkers investigated the interaction type by which *Blautia* is known to be involved in butyrate production [62], which is important for inducing regulatory T cells [63], reducing inflammatory responses [64], and aiding the gut barrier through being an important energy source for enterocytes [65]. Therefore, low relative abundance along with a high copresence interaction rate of *Blautia* could lead to a more inflammatory gut with less healthy enterocytes and thereby an increased risk of GvHD. Meanwhile, the *Citrobacter* genus was described to cause bacteremia in pediatric patients with hematopoietic stem cell transplantation.

A comparison of the network patterns in the GvHD and the no-GvHD subgroups reveal a possible distinction between the real impact of allo-HSCT transplantation and the combined impact of bone marrow graft and GvH disease found in the GvH subgroup. We can hypothesize that the differences in the number of OTUs as well as the members and potential interactions between the GvHD patient and the no-GvHD patient could represent specific markers that could enable us to distinguish whether the dysbiosis is due to the allo-HSCT itself or it is associated to post graft related pathogenesis mechanisms like GvH disease.

Such findings shed light on the importance of and the urgency to clinically restabilize gut microbiota towards a eubiotic status in stem cell transplant recipients in order to prevent drastic outcomes such as infections and GvHD and to help enhance the chances of survival in this fragile population.

### Metabolomics Data

According to our results, the fecal metabolome signature significantly differs between allo-HSCT (allogenic hematopoietic stem cell transplantation) patients and healthy subjects. Furthermore, we reported that allo-HSCT (hematopoietic stem cell transplantation) patients have significant metabolomic changes compared to healthy subjects. Further, we showed that GvHD onset is associated with specific differences in the metabolomics profile compared to patients without GvHD. For instance, all the metabolomics changes could be associated with those of the gut microbiota composition.

Succinic acid and fumaric acid were upregulated in the feces of the allo-HCST recipients compared to the CT group. This increase could be consistently related to the high levels of *Bacteroides*. Both succinic and fumaric acids have been reported to be oncometabolites or endogenous cancer-causing metabolites [66]. In fact, they are intermediate components of the Krebs cycle that significantly increase in cancer tissues [67]. Their accumulation in tumor cells supplies anabolic precursors for tumor growth and induces tumor aggressiveness by causing epigenetic changes such as dysregulation in the anti-metastatic miRNA cluster mir-200ba429 [68]. The accumulation of succinate and fumarate were recently explained by the mutations in the genes coding succinate dehydrogenase (SDH) and fumarate hydratase (FH) enzymes of the TCA cycle and their inactivation [68,69]. Succinate is known to limit the production of anti-inflammatory cytokines, particularly IL-10. Inactivation of SDH inhibits the production of LPS-induced mitochondrial ROS and IL-1β and various proinflammatory genes in macrophages, and enhances the expression of IL-10 and anti-inflammatory genes [70].

Otherwise, lipids levels seem to be altered in the allo-HSCT recipients compared to the healthy group. In our results, we reported increased levels of lipids, especially fatty acids. Recent studies showed a significant increase in serum lipid amounts after allo-HSCT [71,72]. Abnormal amounts of lipids (dyslipidemia) often occur post allo-HSCT and the use of a potent lipid-lowering therapy suggests the role of lipid metabolism in modulating GvHD [73]. Ameliorated lipid synthesis favors the proinflammatory Teff phenotype, whereas lipid oxidation promotes iTreg differentiation, validating the role of fatty acids in GvHD development (Zou and Chen, 2020). Most of the unsaturated fatty acids in our study occurred in higher levels in the patients with GvHD compared to patients without GvHD, which is in accordance with a previously described rise in the plasma of GvHD patients [14]. Polyunsaturated fatty acids (PUFAs) are crucial to tissue homeostasis and cannot be synthesized by the human body, and are frequently obtained through dietary sources. Omega-6 PUFAs are associated with the production of proinflammatory lipids, whereas v-3 PUFAs are metabolized to anti-inflammatory lipid mediators [74]. Polyunsaturated fatty acids are the precursor metabolites of the eicosanoid family, such as leukotriene and prostaglandin (PG) [75]. Leukotriene and prostaglandin are associated with the generation of pro-inflammatory cytokines such as interferon-γ, TNF-α, and IL-17, and gut integrity, respectively. The inactivation of 5-lipoxygenase (5-LO) reduced leukotriene B4 production from arachidonic acid and protected from acute GvHD in an experimental mouse study [76].

Vitamin E is a fat-soluble nutrient. Natural forms of vitamin E, such as alpha-tocopherol (vitamin E), gamma-tocopherols, and delta-tocopherols, have been shown to play a key role in the antioxidant and anti-inflammatory defense systems in the body [77]. Clemens et al. showed that serum levels of α-tocopherol decreased significantly in the first 12 days in post-HSCT patients [78]. Recent data indicated that low serum levels of vitamin E may affect the composition of Firmicutes and Proteobacteria in the intestines [79].

Energy or ATP production in cells requires fundamental cellular processes such as Glycolysis and oxidative phosphorylation [80,81]. Glycolysis interconnects with the Krebs cycle and the pentose phosphate pathway (PPP). Changes in glycolytic activity consequently affect the PPP pathway, which is crucial for nucleotide biogenesis, glutathione reduction, and NADPH regeneration [82]. Studies have shown that glycolysis is required for the appropriate action of alloreactive T cells and GvHD development. Inhibition of glycolysis has been shown to reduce GvHD mortality and morbidity in mouse models [83,84]. This suggests that glycolysis may be a possible therapeutic target to reduce GvHD development.

Besides, we observed a significant diminution in tryptophan metabolites, especially the microbiota-derived compounds (indole-3-methyl acetate and indole) produced by indoleamine 2,3-dioxygenase (IDO), which catabolizes tryptophan into kynurenine [85]. In our study, these two metabolites had low levels in HSCT recipients compared to healthy subjects. Similar results were found by Michonneau et al. in the plasma of allograft allo-HSCT patients [14]. Indole compounds were reported as aryl hydrocarbon receptor (AHR) ligands. Recent studies have demonstrated that IDO induction depends on AHR expression [86]. GvHD development is associated with a decrease in indole compounds that could then limit IDO induction. Alternatively, IDO activity has been linked with GvHD severity in mice and humans [87,88,89]. IDO is capable of diminishing T-cell proliferation and survival at the site of expression, thus reducing GVHD severity in mice models [90]. Gutiérrez-Vázquez and Quintana reported that AHR modulates Th17 response and promotes tolerance through the differentiation and activation of Treg and Tr1 cells [91].

Short-chain fatty acids (SCFA) are the end fermentation products of non-digestible carbohydrates by the gut microbiota. In the current study, SCFA, in particular acetic acid that was detected in the stools of our study population, had low levels in patients with GvHD compared to no GvHD. Recent studies mentioned a direct link between qualitative and quantitative changes of SCFAs and alteration in gut diversity [92]. Acetate is the main metabolite produced by *Bacteroides* and acetate levels have been associated with *Bacteroides* amounts in the gut microbiota [93]. Previously published results showed that acetate functions as a nutritional source for tumors and as a regulator of cancer cell stress [86]. Thus, stopping its recapture by cancer cells may provide an opportunity for therapeutic intervention.

Bile acids are among the crucial metabolites of the intestinal microbiota. It is currently known that the bile acid pathway is mainly involved in promoting the absorption of lipids and fat-soluble nutrients in the intestinal tract and in eliminating body cholesterol [94]. At the onset of GvHD, bile acids have been shown to vary, suggesting the implication of bacterial-derived metabolites in the allogeneic immune response during GvHD. With some studies suggesting a role in pro-inflammatory cytokine production, T-cell activation, and neutrophil recruitment [95,96], and other data suggesting that they could inhibit inflammasome activation [97], the effect of bile acids on immune response and inflammation remains not fully understood.

## 5. Conclusions

The present study provides some insights into the complex cross-talk taking place between gut bacterial diversity and its metabolome signature in allo-HSCT recipients. Interestingly, among the factors that significantly modify patients’ microbiota structure and fecal metabolites is GvHD onset. Several questions should still be addressed in upcoming studies to extend our knowledge of gut microbiome-derived metabolites and their role in determining relevant biological processes in allo-HSCT. Firstly, the metabolomic signature should be more precisely characterized by exploring plasmatic, urinary, and breath metabolites. Then, in our future collaborative studies, we will include larger cohorts to confirm whether GM biomarkers or specific metabolic profiles could be associated with allo-HSCT outcomes.

## Supplementary Materials

The following are available online at https://www.mdpi.com/article/10.3390/microorganisms9091845/s1, Figure S1: Comparison of the mean relative abundance of bacterial operational taxonomic units (OTUs) at the phylum (taxonomic level L2) between study groups. Figure S2: Serial group comparison analysis. Boxplot of significantly different OTUs between control and allo-HSCT individuals. Figure S3: Serial group comparison analysis. Boxplot of significantly different OTUs between allo-HSCT recipients suffering and not suffering from graft-versus-host disease: GvH; no GvH. Figure S4: Total number of positive (co-presence/co-occurrence) and negative (mutual exclusion) relationships between nodes at the genus level within study groups. Figure S5: Pairwise comparisons using the Wilcoxon rank-sum test with continuity correction based on the Benjamini–Hochberg (BH) method between allo-HSCT recipients suffering and not suffering from graft-versus-host disease: GvH; no GvH and the control group. Figure S6: Representative fecal GC/MS spectra with retention time of samples from allo-HSCT patients and healthy volunteers. Figure S7: Heatmap showing the distribution of metabolites among patients and control groups. Figure S8: The permutation test plots for the OPLS-DA models for the classification of allo-HSCT and CT groups with 200 permutation tests. Figure S9: The permutation test plots for the OPLS-DA models for the classification of the GvHD and no-GvHD groups with *n* = 100 permutation tests. Figure S10: Fecal metabolic differences among groups: heatmaps of differential metabolites among the groups. Table S1: Clinical and demographic data of the allo-HSCT patients. Table S2: Alpha diversity indexes of fecal samples of allo-HSCT recipients. Table S3: Mean relative abundances of the seven most abundant bacteria at the genus level present in the fecal specimens of 15 allo-HSCT patients and 18 control subjects. Table S4: Mean relative abundances of the eight most abundant bacteria at the genus level present in the fecal specimens of allo-HSCT recipients suffering or not from graft-versus-host disease. Table S5: Topological properties of networks in fecal communities of healthy and allo-HSCT transplant samples with and without GvH disease. Table S6: Summary of the detected metabolites in the feces of the allo-HSCT and CT groups. Table S7: Biomarkers identified in fecal metabolic profiles of the allo-HSCT group vs. the CT group. Table S8: Selected biomarkers identified in fecal metabolic profiles of the allo-HSCT group vs. the CT group. Table S9: Changes in the metabolite levels in GvHD and non-GvHD patients compared to the CT group.
