# Supplementary material for: High Throughput Analysis Reveals Changes in Gut Microbiota and Specific Fecal Metabolomic Signature in Hematopoietic Stem Cell Transplant Patients
Source: Microorganisms. 2021 Aug 31;9(9):1845. doi: 10.3390/microorganisms9091845 (PMC8469814; doi:10.3390/microorganisms9091845)
Supplement: Supplementary file 1 [file microorganisms-09-01845-s001.zip › microorganisms-1249887-supplementary.pdf]

Table S1: Clinical and demographic data of the allo-HSCT patients.

	Age	*Gender*	*Primary Disease*	*GvHD occurrence*
P1	28	M	AML	GvHD (acute)
P2	12	M	ALL	GvHD (acute)
P3	24	M	ALL	no-GvHD
P4	40	M	MA	no-GvHD
P5	40	M	ALL	GvHD (acute)
P6	17	M	ALL	no-GvHD
P7	24	M	MA	no-GvHD
P8	41	M	AML	no-GvHD
P9	14	M	MA	no-GvHD
P10	23	F	MA	no-GvHD
P11	32	F	AML	no-GvHD
P12	42	F	ALL	no-GvHD
P13	41	M	AML	GvHD (acute)
P14	42	M	AML	no-GvHD
P15	22	F	ALL	no-GvHD
C1	24	F	NA	NA
C2	24	F	NA	NA
C3	24	F	NA	NA
C4	24	F	NA	NA
C5	18	M	NA	NA
C6	25	F	NA	NA
C7	18	F	NA	NA
C8	30	F	NA	NA
C9	30	F	NA	NA
C10	35	F	NA	NA
C11	62	M	NA	NA
C12	30	M	NA	NA
C13	50	M	NA	NA
C14	42	M	NA	NA
C15	35	M	NA	NA
C16	32	M	NA	NA
C17	31	M	NA	NA
C18	62	M	NA	NA

AML: Acute Myeloid Leukemia; **ALL:** Acute Lymphocytic Leukemia **AM:** Medullar Aplasia;

Table S2: Alpha diversity indexes of fecal samples of allo-HSCT recipients

	Number of Reads	*OTU number*	*Richness*	*Simpson*	*Shannon*	*Pielous evenness*
P1	69056	97.11±3.86	*16.53±0.33[a]*	*0.79±0.002 [a]*	*1.95±0.01 [a]*	*0.69±0.004 [a]*
P2	50352	84.33±1.63	23.77±3.71[a]	0.84±0.02 [b]	2.20±0.06 [b]	0.69±0.03 [a]
P3	47901	67.66±0.19	18.03±3.21[b]	0.79±0.01 [b]	1.93±0.05 [c]	0.65±0.01 [a]
P4	20924	48.1±3.46	30.59±4.06[c]	0.64±0.01 [c]	1.31±0.04 [b]	0.51±0.01 [b]
P5	24044	47.83±4.26	34.96±6.01[a]	0.62±0.01 [a]	1.37±0.1 [c]	0.49±0.006 [a]
P6	19427	53.14±1.66	30.99±7.14[d]	0.61±0.009 [a]	1.26±0.06 [d]	0.43±0.04 [b]
P7	18754	60.67±7.05	17.97±2.29[d]	0.71±0.003 [a]	1.69±0.03 [d]	0.60±0.01 [b]
P8	31440	56.41±15.62	17.99±3.05[d]	0.74±0.01 [b]	1.78±0.07 [c]	0.64±0.02 [b]
P9	12788	48.34±0.56	11.89±4.02[d]	0.66±0.1 [d]	1.67±0.23 [e]	0.55±0.06 [c]
P10	19126	47.02±1.88	12.79±4.14[a]	0.90±0.01 [b]	2.69±0.03 [b]	0.78±0.02 [b]
P11	13252	50.44±8.84	15.99±0.87[d]	0.57±0.01 [b]	0.95±0.03 [d]	0.86±0.01 [b]
P12	11854	50.21±2.52	17.99±5.36[d]	0.90±0.01 [b]	2.49±0.09 [c]	0.84±0.01 [a]
P13	13097	52.69±2.27	15.77±5.4[a]	0.47±0.02 [c]	0.12±0.04 [b]	0.06±0.01 [b]
P14	20937	67.7±3.05	15.99±2.3[a]	0.23±0.01 [b]	0.51±0.09 [b]	0.19±0.03 [b]
P15	11072	54.09±5.27	19.94±11.49[d]	0.75±0.01 [b]	1.71±0.04 [c]	0.53±0.06 [a]
C1	35517	278.76±5.01	66.92±3.6 [d]	0.72±0.06 [c]	2.18±0.34 [d]	0.52±0.08 [b]
C2	22288	299.5±1.6	60.58±6.32 [d]	0.84±0.04 [a]	2.63±0.21 [d]	0.64±0.05 [b]
C3	44927	300.63±8.94	55.63±1.84 [d]	0.87±0.02 [c]	2.56±0.11 [d]	0.64±0.02 [b]
C4	57465	292.84±5.03	64.39±2.1 [d]	0.75±0.06 [c]	2.22±0.19 [c]	0.53±0.05 [b]
C5	36469	280.92±7.34	61.62±1.76 [d]	0.91±0.02 [b]	2.96±0.14 [c]	0.72±0.03 [b]
C6	36012	301.47±2.56	70.85±0.54 [b]	0.91±0.02 [a]	2.97±0.1 [c]	0.7±0.02 [b]
C7	31802	306.7±9.72	56.3±0.3 [d]	0.93±0 [b]	3.07±0.04 [c]	0.76±0.01 [a]
C8	42430	304.58±7.41	67.97±1.3 [d]	0.93±0.01 [b]	3.14±0.07 [c]	0.74±0.01 [b]
C9	27857	265.94±2.54	62.65±2.73 [b]	0.94±0 [a]	3.22±0.02 [a]	0.78±0.01 [a]
C10	59253	253.01±3.7	55.18±1.59 [d]	0.9±0.01 [a]	2.77±0.16 [c]	0.69±0.04 [b]
C11	37700	268.99±0.78	57.3±1.76 [d]	0.91±0.01 [a]	2.86±0.06 [c]	0.71±0.01 [b]
C12	11777	278.03±7.11	63±2.52 [d]	0.92±0 [b]	3.09±0.03 [b]	0.75±0.01 [a]
C13	23497	318.02±16.37	57.57±11 .4 [d]	0.73±0.1 [c]	2.1±0.38 [a]	0.52±0.07 [b]
C14	35906	263.91±2.93	75±0.58 [d]	0.93±0.01 [b]	3.24±0.07 [d]	0.75±0.02 [a]
C15	28352	312.86±2.96	52.72±1.88 [a]	0.75±0.01 [c]	1.89±0.03 [b]	0.48±0.01 [b]
C16	40189	293.44±1.64	69.49±1.95 [b]	0.89±0.01 [b]	2.86±0.05 [c]	0.67±0.01 [b]
C17	12749	334.26±12.34	37.24±2.71 [a]	0.56±0.06 [b]	1.49±0.21 [b]	0.41±0.05 [b]
C18	24558	331.07±12.22	52.22±9.76 [a]	0.65±0.1 [c]	1.67±0.32 [b]	0.42±0.06 [b]

Table S3. Mean relative abundances of the seven most abundant bacteria at genus level present in the faecal specimens of 15 allo-HSCT patients and 18 control subjects.

Genera	Relative abundance (Mean ±SE)	
	Control	HSCT
Alistipes	4.93±1.06	0.88±0.29
Akkermansia	1.43±0.57	4.90±2.53
Faecalibacterium	7.82±1.01	1.67±0.59
Klebsiella	0.26±0.14	10.87±3.86
Prevotella.9	21.80±3.71	0.19±0.13
Escherichia.Shigella	0.15±0.15	22.09±3.46
Bacteroides	23.41±2.12	37.76±3.87

Table S4. Mean relative abundances of the eight most abundant bacteria at genus level present in faecal specimens of allo-HSCT recipients suffering or not from graft versus host disease: (GvH; n=4, no GvH; n=11).

Genera		HSCT	
	Control	no GvH	GvH
Alistipes	4.93±1.06	1.00±0.36	0.70±0.15
Veillonella	0.09±0.08	4.86±1.14	3.27±0.49
Akkermansia	1.43±0.57	7.84±3.17	0
Faecalibacterium	7.82±1.01	2.58±0.72	0.17±0.03
Klebsiella	0.26±0.14	16.96±4.75	0.72±0.29
Prevotella.9	21.80±3.71	0.31±0.16	0.003±0.001
Escherichia.Shigella	0.15±0.15	20.71±3.94	24.37±2.66
Bacteroides	23.41±2.82	30.63±3.99	49.65±3.25

Table S5. Topological properties of networks in fecal communities of healthy, and allo-HSCT transplant samples with and without GvH disease.

Sandstorm	Nodes	Edges			Clustering coefficient
		Co-presence*	Mutual exclusion**	Total***	
**Control**	194	599	816	1415	0.270
**GvH**	90	969	916	1885	0.667
**No-GvH**	83	801	615	1416	0.487

* Number of positive interactions (Co-presence / Co-occurrence)

** Number of negative interaction (Mutual exclusion)

*** Total number of edges

Table S6. Summary of the detected metabolites in the feces of allo-HSCT and CT group

Sub-classes	Chemical classes	Metabolites	m/z	Chemical Structure
Aldehydes	Medium-chain aldehyde	4-Oxononanal	43/85/71	MF : $C_9H_{16}O_2$ MW: 156.22 g/mol
Benzenoids	Benzenoids	Benzoic acid	105/77/135	MF : $C_7H_6O_2$ MW: 122.12 g/mol
	Benzenoids	P,P-Dioctyldiphenylamine	322/323/250	MF : $C_{28}H_{43}N$ MW: 393.6 g/mol
	Benzenoids	Mesitylene	105.99/ 120.86/77.98	MF : C_9H_{12} MW: 120.19 g/mol
	Phenylpropanes	2,4-Di-tert-butylphenol	191.99/57.32/41.16	MF : $C_{14}H_{22}O$ MW : 206.17 g/mol
	Phenylpropanes	7,9-Di-tert-butyl-1-oxaspiro[4,5]deca-6,9-diene-2,8-dione	57/205/55	MF : $C_{17}H_{24}O_3$ MW : 276.4 g/mol
Carboxylic acids	Carboxylic acids	5beta-Cholestanone	372.33/43/301	MF : $C_{27}H_{46}O$ MW : 386.7 g/mol
	Monocarboxylic acid	2-Propenoic acid, tridecyl ester	55/43/41	MF : $C_{16}H_{30}O_2$ MW : 254.41 g/mol
	Monocarboxylic acid	Acetic acid	43/45/60	MF : $C_2H_4O_2$ MW : 60.05 g/mol
	Tricarboxylic acids	Triethyl citrate	157/ 203/115	MF : $C_{12}H_{20}O_7$

				MW : 276.28 g/mol
Dicarboxylic acid	Dicarboxylic acid	Succinic acid	72/55/57	MF : $C_4H_6O_4$ MW : 118.09 g/mol
		Glutaric acid, 3-methylbut-2-enyl pentyl ester	115/69/68	MF : $C_{15}H_{26}O_4$ MW : 270.36 g/mol
		Oxalic acid	73/91/55	MF : $C_2H_2O_4$ MW : 90.03 g/mol
		Fumaric acid	115/97/83	MF : $C_4H_4O_4$ MW : 116.07 g/mol
		Malonic acid	59/103/42	MF : $C_3H_4O_4$ MW : 104.06 g/mol
Diamine	Diazines	Thymine glycol	46/89/43	MF : $C_5H_8N_2O_4$ MW : 160.13 g/mol
Food additive	Food additive	2,2'-Bi-1,3-dioxolane, 4,4,4',4',5,5,5',5'-octamethyl-	129/83/101	MF : $C_{14}H_{26}O_4$ MW : 258.35 g/mol
Furans	Furans	2,5-Furandicarboxylic acid	156/139/39	MF : $C_6H_4O_5$ MW : 156.09 g/mol
Hydrocarbons	Alkanes	Nonadecane	57.99 / 43.82 / 71.66	MF : $C_{19}H_{40}$ MW : 268.5 g/mol
		Tritetracontane	57.99 / 71.64 /43.47	MF : $C_{43}H_{88}$ MW : 605.2 g/mol
		Eicosane	55.99/57.77/43.69	MF : $C_{20}H_{40}$ MW : 280.31 g/mol
		Hexadecane	85/99/113	MF : $C_{16}H_{34}$ MW : 226.44 g/mol
		Octadecane	57/43/71	MF : $C_{18}H_{38}$ MW : 254.5 g/mol
		Heptadecane	57.99 / 43.65 / 71.64	MF : $C_{17}H_{36}$ MW :240.5 g/mol
		cis-9-Tricosene	55/97/83	MF : $C_{23}H_{46}$ MW : 322.6 g/mol
	Alkenes	1-Nonacosene	57.99/43.95/55.91	MF : $C_{29}H_{58}$ MW :406.45 g/mol
	Hydrocarbons	Carbonic acid, eicosyl vinyl ester	57/71/85	MF : $C_{23}H_{44}O_3$

				MW : 368.6 g/mol
	Unsaturated aliphatic hydrocarbon	1-Pentadecene	97/111/83	MF : $C_{15}H_{30}$<br>MW : 210.4 g/mol
Indoles	Indoles	Indole	117.05/89.02/101.03	MF : C_8H_7N<br>MW : 117.15 g/mol
		Indole-3-methyl acetate	130/189/131	MF: $C_{11}H_{11}NO_2$<br>MW : 189.21 g/mol
Ketones	Ketones	2-Tridecanone	58/43/71	MF : $C_{13}H_{26}O$<br>MW : 198.34 g/mol
Lipids	iso-fatty acids	Methyl 13-methyltetradecanoate	75/87.65/55.24	MF : $C_{16}H_{32}O_2$<br>MW : 256.24 g/mol
	Long-chain fatty acid	17-Octadecynoic acid	55/81/67	MF : $C_{18}H_{32}O_2$<br>MW : 280.4 g/mol
		12-Methyltridecanoic acid	74.99/87.69/43.47	MF: $C_{15}H_{30}O_2$<br>MW: 242.25 g/mol
		Oleic acid, methyl ester	55.99/69.66/74.54	MF: $C_{19}H_{36}O_2$<br>MW: 296.27 g/mol
		16-Methylheptadecanoic acid	74.99/87.76/75.23	MF: $C_{19}H_{38}O_2$<br>MW: 298.29 g/mol
		Palmitoleic acid	237/219/171	MF : $C_{16}H_{30}O_2$<br>MW : 254.41 g/mol
		17-Methyloctadecanoic acid	73/43/89	MF : $C_{19}H_{38}O_2$<br>MW : 298.5 g/mol
		Z-8-Methyl-9-tetradecenoic acid	55/41/43	MF : $C_{15}H_{28}O_2$<br>MW : 240.38 g/mol
		Tetracosanoic acid	117/129/132	MF : $C_{24}H_{48}O_2$<br>MW : 368.6 g/mol
	Phytosterols	beta-Sitosterol	43.99/ 55.35/41.33	MF : $C_{29}H_{50}O$<br>MW: 414.39 g/mol
		Stigmasterol	55.99/83.69/81.64	MF : $C_{29}H_{48}O$<br>MW: 412.37 g/mol
		Campesterol	43.99/55.62/41.47	MF: $C_{28}H_{48}O$<br>MW: 400.37 g/mol
	Sterol	Cholesta-4,6-dien-3-ol, (3.beta,)	43.99/143.7/135.63	MF: $C_{27}H_{44}O$

				MW: 384.34 g/mol
		Cholesterol	43.99/55.89/57. 74	MF : $C_{27}H_{46}O$ MW: 386.35 g/mol
		Lathosterol	43.99/386.89/255.73	MF : $C_{27}H_{46}O$ MW: 386.36 g/mol
		Cholest-5-en-3-ol	43/386/57	MF : $C_{27}H_{46}O$ MW: 386.7 g/mol
	Steroid esters	Cholesterol Myristate	43/57/81	MF : $C_{41}H_{72}O_2$ MW: 597 g/mol
	steroid derivative	Stigmastanol	43.99/107.72/215.7	MF : $C_{29}H_{52}O$ MW : 416.4 g/mol
	Stigmastanes	Clionasterol	43.99/55.67/57.56	MF : $C_{29}H_{50}O$ MW : 414.39 g/mol
	Cholestane steroids	Coprostanol	43.99/55.94/215.87	MF : $C_{27}H_{48}O$ MW : 388.37 g/mol
		Cholestenone	124/43/55	MF : $C_{27}H_{44}O$ MW : 384.6 g/mol
	Ergostane steroids	Ergosterol	142/118/80	MF : $C_{28}H_{44}O$ MW : 396.6 g/mol
	Fatty acid methyl esters	methyl palmitate	75/87.72/43.325	MF : $C_{17}H_{34}O_2$ MW : 270.256 g/mol
		13-Octadecenoic acid, methyl ester	55.99/41.63/69.54	MF : $C_{19}H_{36}O_2$ MW : 296.27 g/mol
	Fatty acid esters	2-Hexenyl valerate	85/57/41	MF : $C_{11}H_{20}O_2$ MW : 184.27 g/mol
		Methyl 3-phenylpropanoate	104/91/105	MF : $C_{10}H_{12}O_2$ MW : 164.2 g/mol
		Ethyl Oleate	43.99/55.68/69.67	MF : $C_{20}H_{38}O_2$ MW : 310.29 g/mol
		Diethylhexyl adipate	129/371/259	MF: $C_{22}H_{42}O_4$ MW: 370.6 g/mol
	Fatty alcohol	Cetyl alcohol	56/69/83	MF: $C_{16}H_{34}O$ MW: 242.26 g/mol
		(Z)-5-Octen-1-ol	67/41/68	MF: $C_8H_{16}O$ MW: 128.21 g/mol

	Fatty alcohol esters	Lauryl fluoroacetate	69/55/83	MF: $C_{14}H_{27}FO_2$ MW: 246.36 g/mol
		cis-5-Decen-1-yl acetate	43/81/67	MF: $C_{12}H_{22}O_2$ MW: 198.3 g/mol
Naphthalenes	Naphthalenes	1,4-Naphthoquinone	217.99/232.91/189.44/43.27	MF : $C_{12}H_8O_5$ MW : 232.04 g/mol
Other	Unknown	Isobutyl 2-methylpentyl carbonate	57/84/85	MF: $C_{11}H_{22}O_3$ MW: 202.29 g/mol
		3,9-Diethyl-6-tridecanol	69/83/55	MF: $C_{17}H_{36}O$ MW: 256.5 g/mol
		Methyl 7,11,14-eicosatrienoate	68/81.67/79.59	MF: $C_{21}H_{36}O_2$ MW: 320.27 g/mol
		Ethyl 9,12-hexadecadienoate	67/81/55	MF : $C_{18}H_{32}O_2$ MW : 280.4 g/mol
Phenols	Phenols	m-Cresol	108.99/107.95/79.35	MF : C_7H_8O MW : 108.06 g/mol
		2-Methoxy-4-vinylphenol	150/135/107	MF: $C_9H_{10}O_2$ MW: 150.17 g/mol
Terpenoids	Monoterpenoids	Camphor	95/69/55	MF: $C_{10}H_{16}O$ MW: 152.23 g/mol
	Terpenoids	1,2-Epoxy-1,2,7,7',8,8',11,12-octahydro-psi,psi-carotene	544/473/69	MF: $C_{40}H_{64}O$ MW: 560.9 g/mol
	Carotenoid	Squalene	69.06/81.06/41.03	MF : C30H50 MW: 410.7 g/mol
	Diterpenoid	Phytol	143/73/75	MF: $C_{20}H_{40}O$ MW: 296.5 g/mol
Vitamins	Tocopherols	Gamma-Tocopherol	151.99/416.72/417.21	MF :$C_{28}H_{48}O_2$ MW : 416.37 g/mol
		Vitamin E	165.99/430.79/164.31	MF :$C_{29}H_{50}O_2$ MW : 430.38 g/mol

MF: Molecular Formula, **MW:** Molecular weight

Table S7. Biomarkers identified in fecal metabolic profiles of allo-HSCT group vs CT group. aArea under the receiver operating characteristic (ROC) curve of the biomarkers; bSensitivity and cSpecificity were calculated from the ROC curve

Name	VIP	FDR	FC	AUC	Sensitivity	Specificity
P,P-Dioctyldiphenylamine	1,098	0.0013207	20.238	1.0	1	1
Fumaricacid	1,55	0.0012967	0.013105	1.0	1	1
Squalene	2,119	0.019419	0.033063	1.0	1	1
Indole-3-methyl acetate	1,245	9.4356E-4	0.21604	1.0	1	1
Lathosterol	1,189	8.0299E-4	6.6603	1.0	1	1
Tetracosanoicacid	2,085	0.0098185	0.046407	1.0	1	1
Vitamin E	2,243	0.011124	0.082115	0.998	0,99	1
Succinicacid	2,227	0.012497	0.12823	0.994	0,994	1
m-Cresol	2,532	0.009682	12.958	0.978	0,956	1
Z-8-Methyl-9-tetradecenoic acid	2,143	3.7065E-4	9.8334	0.937	0,874	1
2-Hexenyl valerate	4,318	1.0586E-4	3.5335	0.934	0,938	0,93
Methyl 3-phenylpropanoate	1,421	3.7674E-4	16.671	0.933	0,866	1
Cholestenone	2,402	1.5487E-4	0.41437	0.929	0,858	1
Phytol	1,587	5.9967E-4	10.731	0.902	0,804	1
beta-Sitosterol	2,217	6.4757E-4	0.47736	0.877	0,854	0,9
3,9-Diethyl-6-tridecanol	1,711	3.7674E-4	10.123	0.871	0,742	1
Cholesterol	2,083	0,0043436	7.0209	0.868	0,736	1
5beta-Cholestanone	1,961	0.001398	3.0618	0.851	0,902	0,8
Campesterol	1,549	5.0494E-4	2.8208	0.846	0,702	0,99
16-Methylheptadecanoic acid	1,774	3.5182E-5	39.607	0.827	0,772	0,972
Aceticacid	1,899	0.0017588	3.4924	0.789	0,63	0,948
Lathosterol	3,874	0.040879	2.2564	0.773	0,696	0,85
17-Methyloctadecanoic acid	1,431	0.036001	892.23	0.745	0,592	0,898
Indole	5,571	0,006894	64.862	0.759	0,638	0,88

FC: Fold Change;FDR: False Discovery Rates;VIP: The variable importance in the projection; AUC: area under the curve

Table S8. Biomarkers identified in fecal metabolic profiles of allo-HSCT group vs CT group. aArea under the receiver operating characteristic (ROC) curve of the biomarkers; bSensitivity and cSpecificity were calculated from the ROC curve

Name	VIP	FDR	FC	AUC	Sensitivity	Specificity
(9Z) -Octadecenoicacid	3,28	8.1257E-4	9.2694	0.977	0,954	1,0
Cholesterol	1,82	0.017742	3.2579	0.85	0,8	0,9
campesterol	1,65	0.025	5.0965	0,784	0,718	0,85
Lathosterol	1,49	0.029516	2.2313	0,756	0,612	0,9

FC: Fold Change;FDR: False Discovery Rates;VIP: The variable importance in the projection; AUC: area under the curve

Table S9. Changes of the metabolites' levels in GvHD and Non-GvHD patients compared to CT group

Compound	Non_GvHD vs CT group	GvHD vs CT group
Succinicacid	0,00038↑	0,013↑
17-Octadecynoic acid	0,0153↓	0,0014↑
12-Methyltridecanoic acid	0,0181↓	0,0294↑
Methyl 3-phenylpropanoate	0,0018↓	0,026↑
Coprostanol	8,48E-05↓	0,0002↑
Lathosterol	0,0369↓	0,0211↑
Aceticacid	0,0139↓	0,0122↓
Stigmastanol	0,00016↑	0,0002↑
17-Methyloctadecanoic acid	0,0054↓	0,193
Ergosterol	0,0094↑	0,132
2-Methoxy-4-vinylphenol	0,0083↓	0,0344↑
Squalene	0,0003↑	0,254
Vitamin E	0,0024↓	0,0724
Indole-3-methyl acetate	0,0114↓	0,111
7,9-Di-tert-butyl-1-oxaspiro[4,5]deca-6,9-diene-2,8-dione	6,71E-05↓	0,389
m-Cresol	0,0005↓	0,0852
2,4-Di-tert-butylphenol	0,118	0,033↓

↓Significant decrease in Non-GvHD or GvHD patients compared to Healthy controls ($p < 0.05$) ;

↑ Significant decrease in Non-GvHD or GvHDpatients compared to Healthy controls ($p < 0.05$).
